# Bis[(2*R*,6*S*)-4-(5-amino-3-carb­oxy-1-cyclo­propyl-6,8-difluoro-4-oxo-1,4-dihydro­quinolin-7-yl)-2,6-dimethyl­piperazin-1-ium] sulfate penta­hydrate

**DOI:** 10.1107/S160053681104757X

**Published:** 2011-11-19

**Authors:** Tao Li, Lin Yang, Yuan Cheng Wang, Qiang Lian

**Affiliations:** a School of Life Sciences, Fujian Agriculture and Forestry University, Fuzhou, Fujian 350002, People’s Republic of China

## Abstract

The title compound, C_19_H_23_F_2_N_4_O_3_^+^·0.5SO_4_^2−^·2.5H_2_O, an anti­bacterial fluoro­quinolone, crystallized as a racemic twin (major twin component = 0.633) in the chiral space group *P*1. The asymmetric unit contains two sparfloxacinium cations, one sulfate anion and five mol­ecules of water of solvation. The bond lengths and angles of both cations are almost identical. The quinoline ring systems in the cations are essentially planar, the mean deviations from the best plane being 0.045 (2) and 0.054 (2) Å and make π–π inter­actions with each other [centroid–centroid distances of 3.692 (4) Å and 3.744 (4) Å]. The crystal structure features inter­molecular O—H⋯O, O—H⋯S, N^+^—H⋯O, N^+^—H⋯S and N—H⋯O hydrogen bonds together with intra­molecular O—H⋯O and N—H⋯O hydrogen bonds. As a result, a three-dimensional supra­molecular structure is observed.

## Related literature

For the biological activity of sparfloxacin compounds, see: Truffot-Pernot *et al.* (1993 ▶). For structures containing sparfloxacin, see: Sivalakshmidevi *et al.* (2000 ▶); Shingnapurkar *et al.* (2007 ▶); Kalliopi *et al.* (2000 ▶). 
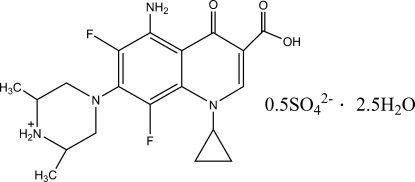


## Experimental

### 

#### Crystal data

2C_19_H_23_F_2_N_4_O_3_^+^·SO_4_^2−^·5H_2_O
*M* *_r_* = 972.97
Triclinic, 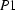

*a* = 7.1961 (3) Å
*b* = 9.6892 (4) Å
*c* = 15.6136 (5) Å
α = 84.760 (6)°
β = 83.045 (5)°
γ = 88.619 (5)°
*V* = 1076.03 (7) Å^3^
*Z* = 1
Mo *K*α radiation
μ = 0.17 mm^−1^
*T* = 173 K
0.20 × 0.20 × 0.20 mm

#### Data collection

Rigaku Mercury CCD/AFC diffractometer
Absorption correction: multi-scan (*CrystalClear*; Rigaku, 2007 ▶) *T*_min_ = 0.966, *T*_max_ = 0.966
8268 measured reflections
6614 independent reflections
5665 reflections with *I* > 2σ(*I*)
*R*_int_ = 0.027

#### Refinement

*R*[*F* ^2^ > 2σ(*F* ^2^)] = 0.039
*wR*(*F* ^2^) = 0.086
*S* = 0.97
6614 reflections
620 parameters
3 restraints
H-atom parameters constrained
Δρ_max_ = 0.25 e Å^−3^
Δρ_min_ = −0.28 e Å^−3^

### 

Data collection: *CrystalClear* (Rigaku, 2007 ▶); cell refinement: *CrystalClear*; data reduction: *CrystalClear*; program(s) used to solve structure: *SHELXS97* (Sheldrick, 2008 ▶); program(s) used to refine structure: *SHELXL97* (Sheldrick, 2008 ▶); molecular graphics: *SHELXTL* (Sheldrick, 2008 ▶); software used to prepare material for publication: *SHELXL97*.

## Supplementary Material

Crystal structure: contains datablock(s) global, I. DOI: 10.1107/S160053681104757X/im2333sup1.cif

Structure factors: contains datablock(s) I. DOI: 10.1107/S160053681104757X/im2333Isup2.hkl

Supplementary material file. DOI: 10.1107/S160053681104757X/im2333Isup3.cml

Additional supplementary materials:  crystallographic information; 3D view; checkCIF report

## Figures and Tables

**Table 1:** Hydrogen-bond geometry (Å, °)

*D*—H⋯*A*	*D*—H	H⋯*A*	*D*⋯*A*	*D*—H⋯*A*
N1—H5⋯O8^i^	0.92	1.81	2.724 (3)	170
N1—H5⋯S1^i^	0.92	2.99	3.860 (3)	158
N1—H13⋯O10^ii^	0.84	2.12	2.799 (3)	138
N1—H13⋯O12^ii^	0.84	2.60	3.257 (4)	135
N3—H2⋯O3	0.86	1.97	2.670 (3)	138
N3—H10⋯O11	0.89	2.07	2.965 (3)	174
N5—H8⋯O12^iii^	0.90	1.80	2.687 (3)	169
N5—H12⋯O8	0.90	1.83	2.722 (3)	174
N5—H12⋯S1	0.90	2.80	3.628 (3)	154
N7—H1⋯O6	0.90	2.00	2.673 (3)	131
N7—H4⋯O15	0.93	2.10	2.997 (3)	162
O2—H6⋯O3	0.88	1.70	2.523 (3)	156
O5—H9⋯O6	0.96	1.64	2.543 (3)	156
O11—H18⋯O13^iv^	0.86	2.06	2.918 (3)	177
O11—H14⋯O10^iv^	0.82	2.04	2.846 (3)	169
O12—H12*D*⋯O7	0.93	1.81	2.680 (3)	155
O12—H12*D*⋯S1	0.93	2.68	3.479 (2)	145
O12—H12*C*⋯O13	0.94	1.92	2.748 (3)	146
O13—H13*C*⋯O14	0.84	1.90	2.725 (3)	169
O13—H13*D*⋯O4^v^	0.90	1.95	2.774 (3)	153
O14—H14*C*⋯O1	1.00	1.89	2.834 (3)	157
O14—H14*D*⋯O9^vi^	0.89	1.83	2.715 (3)	170
O15—H15*A*⋯O7^vii^	0.91	1.85	2.748 (3)	168
O15—H15*A*⋯S1^vii^	0.91	2.87	3.680 (2)	150
O15—H15*B*⋯O1^iii^	0.89	2.29	2.979 (3)	134

Symmetry codes: (i) 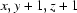; (ii) 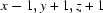; (iii) 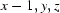; (iv) 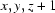; (v) 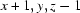; (vi) 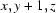; (vii) 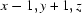.

**Fig. 1.**
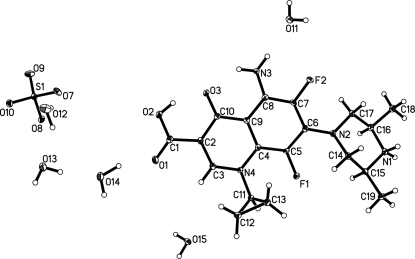
Molecular structure of one sparfloxacinium cation, sulfate and water molecules, displacement ellipsoids are drawn at the 30% probability level (the second cation was omitted for clarity).

**Fig. 2.**
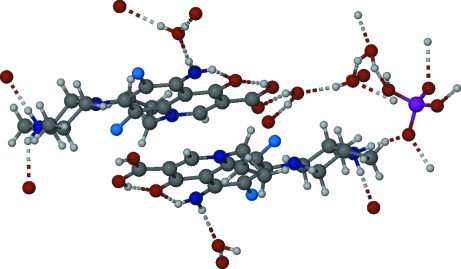
Intramolecular and intermoleclar hydrogen bonds (dashed lines) in the structure of the title compound.

## supplementary crystallographic information

### Comment

Sparfloxacin belongs to the fourth-generation fluorinated quinolone antimicrobial agents, which have been widely used in the treatment of infections (Truffot-Pernot *et al.*, 1993). Generally the poor solubility of a drug will decrease it's bioavailability. Since sparfloxacin shows a solubility-limited bioavailability, a challenging task in the product development is to improve its solubility. Indeed, a widely accepted approach to overcome poor solubility or inadequate material properties of sparfloxacin is the preparation of the respective salts with protonated sparfloxacin cations. Several structures containing sparfloxacin have been reported, including several salts and metal complexes (Sivalakshmidevi *et al.*, 2000; Shingnapurkar *et al.*, 2007; Kalliopi *et al.*, 2000). Here we report the crystal and molecular structure of sparfloxacin hemisulfate 2.5-hydrate.

The title compound crystallizes in the triclinic space group P1 with two sparfloxacinium cations, one sulfate anion and five hydrate molecules in the asymmetric unit. (Fig. 1). The bond distances and angles are in good agreement with those in *cis*-5-amino-1-cyclopropyl-7- (3,5-dimethylpiperazin-1-yl)-6,8- difluoro-1,4-dihydro-4-oxoquinoline-3-carboxylic acid trihydrate (Sivalakshmidevi *et al.*, 2000). The carboxyl groups in both cations are coplanar with the respective quinolyl moiety, while the planes composed of the cyclopropyl groups are inclined at 70.1 (1)° and 71.9 (1)° with respect to the quinolyl rings. The C—O and C═O bond average distances of the carboxylic acid groups of sparfloxacin molecule are of 1.323 (4) Å and 1.219 (4) Å, respectively. The piperazinium ring adopts a chair conformation. Crystal packing is stabilized by π-π stacking interactions of quinoline rings, in which the N4 ring (N4/C2—C10) stacks with the N8 ring (N8/C21—C29) showing centroid-centroid separations of 3.692 (4) Å and 3.744 (4) Å. Due to the presence of a lot of potential hydrogen bond donor and acceptor sites, numerous intramolecular and intermolecular hydrogen bonds are observed in the crystal structure. (Table 1, Fig. 2)

### Experimental

In an attempt to synthesize a vanadium complex a mixture of sparfloxacin (0.4 mmol, 157 mg), vanadyl sulfate hydrate (0.2 mmol, 36 mg) and water (30 ml) was heated to reflux at 100 ° for 4 h. The resulting green crystals were collected through filtration. Anal. calc. for C_38_H_56_F_4_N_8_O_15_S: C, 46.91; H, 5.80; N,11.52; O, 24.67%; Found: C, 46.72; H, 5.83; N,11.51; O, 24.63%. IR (KBr pellet) [cm^-1^]: 3418(*w*), 1715(*m*), 1633(*vs*), 1590(*w*), 1515(*m*), 1439(*vs*), 1384(*w*), 1300(*m*), 1320(*m*), 1112(*m*), 1030(*w*), 960(*w*), 900(*w*), 870(*w*).

### Refinement

H atoms were were located in difference maps and were refined using a riding model with bond lengths C—H = 0.95–1.00 Å, N—H = 0.84–0.93 Å and O—H = 0.82–1.00 Å). *U*_iso_(H) values were fixed at 1.5*U*_eq_ of the parent atom for methyl H atoms and 1.2*U*_eq_ of the parent atom for all other cases. The highest electron-density peak is situated 0.61 Å from C21 and the deepest hole 0.69 Å from S1.

### Crystal data

**Table:**

2C_19_H_23_F_2_N_4_O_3_^+^·SO_4_^2^^−^·5H_2_O	*Z* = 1
*M**_r_* = 972.97	*F*(000) = 512
Triclinic, *P*1	*D*_x_ = 1.501 Mg m^−^^3^
Hall symbol: p 1	Mo *K*α radiation, λ = 0.71073 Å
*a* = 7.1961 (3) Å	Cell parameters from 3371 reflections
*b* = 9.6892 (4) Å	θ = 2.1–27.5°
*c* = 15.6136 (5) Å	µ = 0.17 mm^−^^1^
α = 84.760 (6)°	*T* = 173 K
β = 83.045 (5)°	Prism, green
γ = 88.619 (5)°	0.20 × 0.20 × 0.20 mm
*V* = 1076.03 (7) Å^3^	

### Data collection

**Table:**

Rigaku Mercury CCD/AFC diffractometer	6614 independent reflections
Radiation source: fine-focus sealed tube	5665 reflections with *I* > 2σ(*I*)
graphite	*R*_int_ = 0.027
φ and ω scans	θ_max_ = 25.5°, θ_min_ = 2.4°
Absorption correction: multi-scan (*CrystalClear*; Rigaku, 2007)	*h* = −8→8
*T*_min_ = 0.966, *T*_max_ = 0.966	*k* = −11→11
8268 measured reflections	*l* = −18→18

### Refinement

**Table:**

Refinement on *F*^2^	Primary atom site location: structure-invariant direct methods
Least-squares matrix: full	Secondary atom site location: difference Fourier map
*R*[*F*^2^ > 2σ(*F*^2^)] = 0.039	Hydrogen site location: inferred from neighbouring sites
*wR*(*F*^2^) = 0.086	H-atom parameters constrained
*S* = 0.97	*w* = 1/[σ^2^(*F*_o_^2^) + (0.0396*P*)^2^] where *P* = (*F*_o_^2^ + 2*F*_c_^2^)/3
6614 reflections	(Δ/σ)_max_ < 0.001
620 parameters	Δρ_max_ = 0.25 e Å^−^^3^
3 restraints	Δρ_min_ = −0.28 e Å^−^^3^

### Special details

**Table:**

Geometry. All e.s.d.'s (except the e.s.d. in the dihedral angle between two l.s. planes) are estimated using the full covariance matrix. The cell e.s.d.'s are taken into account individually in the estimation of e.s.d.'s in distances, angles and torsion angles; correlations between e.s.d.'s in cell parameters are only used when they are defined by crystal symmetry. An approximate (isotropic) treatment of cell e.s.d.'s is used for estimating e.s.d.'s involving l.s. planes.
Refinement. Refinement of *F*^2^ against ALL reflections. The weighted *R*-factor *wR* and goodness of fit *S* are based on *F*^2^, conventional *R*-factors *R* are based on *F*, with *F* set to zero for negative *F*^2^. The threshold expression of *F*^2^ > σ(*F*^2^) is used only for calculating *R*-factors(gt) *etc*. and is not relevant to the choice of reflections for refinement. *R*-factors based on *F*^2^ are statistically about twice as large as those based on *F*, and *R*- factors based on ALL data will be even larger.

### Fractional atomic coordinates and isotropic or equivalent isotropic displacement parameters (Å^2^)

**Table:**

	*x*	*y*	*z*	*U*_iso_*/*U*_eq_	
S1	0.61237 (10)	0.18748 (8)	0.24069 (5)	0.01658 (17)	
F1	0.2557 (2)	1.11521 (17)	0.86005 (11)	0.0187 (4)	
F3	−0.0073 (2)	0.84613 (17)	0.47689 (10)	0.0228 (4)	
F2	0.4920 (2)	0.74197 (18)	1.03412 (11)	0.0229 (4)	
F4	0.2141 (2)	0.46692 (17)	0.65128 (10)	0.0189 (4)	
O1	0.5379 (3)	0.7840 (2)	0.48993 (13)	0.0236 (5)	
O2	0.6093 (3)	0.5909 (2)	0.56733 (14)	0.0221 (5)	
O3	0.5740 (3)	0.5853 (2)	0.73035 (13)	0.0186 (5)	
O4	−0.0362 (3)	0.7957 (2)	1.02049 (13)	0.0226 (5)	
O5	−0.1285 (3)	0.9874 (2)	0.94714 (14)	0.0232 (5)	
O6	−0.1107 (3)	0.9932 (2)	0.78319 (13)	0.0189 (5)	
O7	0.6739 (3)	0.2460 (2)	0.31751 (12)	0.0225 (5)	
O8	0.4349 (3)	0.2602 (2)	0.22288 (13)	0.0199 (5)	
O9	0.5785 (3)	0.0393 (2)	0.26000 (15)	0.0293 (5)	
O10	0.7536 (3)	0.2164 (2)	0.16511 (12)	0.0214 (5)	
O11	0.7052 (3)	0.4663 (2)	1.05899 (14)	0.0248 (5)	
O12	0.9648 (3)	0.4140 (3)	0.26693 (15)	0.0333 (6)	
O13	0.8398 (3)	0.6479 (2)	0.17674 (14)	0.0280 (5)	
O14	0.7095 (3)	0.7864 (2)	0.31659 (14)	0.0289 (5)	
O15	−0.3039 (3)	1.0681 (2)	0.46352 (13)	0.0251 (5)	
N1	0.1422 (4)	1.1751 (3)	1.14629 (16)	0.0162 (6)	
N2	0.3324 (4)	1.0124 (3)	1.02055 (16)	0.0184 (6)	
N3	0.5454 (4)	0.5882 (3)	0.90227 (16)	0.0189 (6)	
N4	0.4051 (3)	0.9932 (2)	0.70917 (15)	0.0137 (5)	
N5	0.2724 (3)	0.4163 (2)	0.34861 (15)	0.0168 (6)	
N6	0.1588 (4)	0.5747 (3)	0.48944 (15)	0.0171 (6)	
N7	−0.0781 (4)	0.9943 (3)	0.61074 (16)	0.0185 (6)	
N8	0.0567 (3)	0.5850 (2)	0.80159 (15)	0.0144 (6)	
C1	0.5499 (4)	0.7211 (3)	0.5606 (2)	0.0173 (7)	
C2	0.5003 (4)	0.7852 (3)	0.64338 (19)	0.0151 (7)	
C3	0.4458 (4)	0.9225 (3)	0.63933 (19)	0.0159 (7)	
H3	0.4365	0.9697	0.5839	0.019*	
C4	0.4118 (4)	0.9270 (3)	0.79258 (18)	0.0132 (7)	
C5	0.3558 (4)	0.9936 (3)	0.86610 (19)	0.0131 (6)	
C6	0.3824 (4)	0.9379 (3)	0.95065 (19)	0.0149 (7)	
C7	0.4522 (4)	0.8036 (3)	0.95596 (19)	0.0168 (7)	
C8	0.4906 (4)	0.7221 (3)	0.88655 (19)	0.0151 (7)	
C9	0.4738 (4)	0.7862 (3)	0.80152 (19)	0.0135 (6)	
C10	0.5200 (4)	0.7110 (3)	0.72568 (19)	0.0138 (6)	
C11	0.3807 (4)	1.1442 (3)	0.69580 (19)	0.0152 (7)	
H11	0.2495	1.1813	0.7038	0.018*	
C12	0.5164 (5)	1.2229 (3)	0.6292 (2)	0.0210 (7)	
H12A	0.6141	1.1691	0.5958	0.025*	
H12B	0.4689	1.3061	0.5966	0.025*	
C13	0.5295 (4)	1.2297 (3)	0.7241 (2)	0.0182 (7)	
H13A	0.6352	1.1802	0.7489	0.022*	
H13B	0.4901	1.3171	0.7496	0.022*	
C14	0.3684 (4)	1.1602 (3)	1.01852 (19)	0.0174 (7)	
H14A	0.4069	1.1983	0.9581	0.021*	
H14B	0.4716	1.1747	1.0532	0.021*	
C15	0.1925 (4)	1.2349 (3)	1.05516 (18)	0.0167 (7)	
H15	0.0886	1.2168	1.0207	0.020*	
C16	0.1175 (4)	1.0209 (3)	1.15505 (19)	0.0176 (7)	
H16	0.0038	0.9991	1.1280	0.021*	
C17	0.2866 (4)	0.9490 (3)	1.10943 (18)	0.0173 (7)	
H17A	0.3953	0.9559	1.1420	0.021*	
H17B	0.2596	0.8495	1.1081	0.021*	
C18	0.0877 (4)	0.9714 (3)	1.25073 (19)	0.0195 (7)	
H18A	0.2003	0.9892	1.2774	0.029*	
H18B	0.0628	0.8718	1.2575	0.029*	
H18C	−0.0193	1.0213	1.2790	0.029*	
C19	0.2163 (4)	1.3911 (3)	1.0537 (2)	0.0201 (7)	
H19A	0.1000	1.4329	1.0796	0.030*	
H19B	0.2453	1.4308	0.9936	0.030*	
H19C	0.3188	1.4100	1.0867	0.030*	
C20	−0.0654 (4)	0.8576 (3)	0.9520 (2)	0.0175 (7)	
C21	−0.0290 (4)	0.7921 (3)	0.86875 (19)	0.0147 (7)	
C22	0.0190 (4)	0.6558 (3)	0.87196 (19)	0.0154 (7)	
H22	0.0264	0.6074	0.9272	0.018*	
C23	0.0566 (4)	0.6546 (3)	0.71849 (19)	0.0134 (7)	
C24	0.1170 (4)	0.5900 (3)	0.64409 (19)	0.0143 (7)	
C25	0.0979 (4)	0.6487 (3)	0.56021 (19)	0.0152 (7)	
C26	0.0255 (4)	0.7821 (3)	0.55586 (17)	0.0145 (7)	
C27	−0.0195 (4)	0.8598 (3)	0.62513 (19)	0.0142 (7)	
C28	−0.0051 (4)	0.7952 (3)	0.70999 (19)	0.0134 (6)	
C29	−0.0537 (4)	0.8683 (3)	0.78686 (19)	0.0145 (7)	
C30	0.0722 (4)	0.4335 (3)	0.81326 (19)	0.0153 (7)	
H30	0.2017	0.3928	0.8075	0.018*	
C31	−0.0717 (4)	0.3567 (3)	0.87605 (19)	0.0189 (7)	
H31A	−0.1696	0.4117	0.9083	0.023*	
H31B	−0.0313	0.2709	0.9085	0.023*	
C32	−0.0749 (4)	0.3550 (3)	0.77976 (19)	0.0175 (7)	
H32A	−0.0368	0.2681	0.7533	0.021*	
H32B	−0.1751	0.4089	0.7531	0.021*	
C33	0.0929 (4)	0.4314 (3)	0.48901 (19)	0.0179 (7)	
H33A	−0.0253	0.4328	0.4624	0.022*	
H33B	0.0680	0.3886	0.5492	0.022*	
C34	0.2388 (4)	0.3472 (3)	0.43858 (18)	0.0167 (7)	
H34	0.3577	0.3479	0.4658	0.020*	
C35	0.3365 (4)	0.5622 (3)	0.34571 (19)	0.0163 (7)	
H35	0.4604	0.5625	0.3686	0.020*	
C36	0.1950 (4)	0.6453 (3)	0.40178 (19)	0.0179 (7)	
H36A	0.2441	0.7390	0.4050	0.021*	
H36B	0.0766	0.6557	0.3753	0.021*	
C37	0.3584 (4)	0.6241 (3)	0.25213 (19)	0.0212 (7)	
H37A	0.4465	0.5671	0.2171	0.032*	
H37B	0.4063	0.7185	0.2490	0.032*	
H37C	0.2366	0.6266	0.2299	0.032*	
C38	0.1820 (4)	0.1980 (3)	0.43606 (19)	0.0244 (8)	
H38A	0.0778	0.1953	0.4013	0.037*	
H38B	0.1431	0.1575	0.4951	0.037*	
H38C	0.2886	0.1450	0.4101	0.037*	
H1	−0.1300	1.0360	0.6569	0.027 (10)*	
H2	0.5807	0.5485	0.8562	0.027 (10)*	
H10	0.5867	0.5545	0.9519	0.014 (8)*	
H4	−0.1254	1.0283	0.5602	0.061 (14)*	
H5	0.2355	1.1977	1.1777	0.023 (9)*	
H6	0.6026	0.5645	0.6229	0.054 (13)*	
H13C	0.7892	0.6957	0.2157	0.045 (12)*	
H8	0.1637	0.4071	0.3267	0.047 (12)*	
H9	−0.1305	1.0152	0.8867	0.072 (15)*	
H18	0.7401	0.5202	1.0947	0.058 (13)*	
H12	0.3331	0.3680	0.3072	0.067 (14)*	
H13	0.0447	1.2048	1.1748	0.027 (10)*	
H14	0.7296	0.3913	1.0841	0.083 (18)*	
H15A	−0.2943	1.1247	0.4134	0.087 (17)*	
H15B	−0.3333	0.9936	0.4382	0.076 (16)*	
H14C	0.6764	0.7678	0.3807	0.072 (14)*	
H12D	0.8447	0.3787	0.2790	0.15 (3)*	
H13D	0.8804	0.7179	0.1378	0.15 (3)*	
H14D	0.6538	0.8657	0.3005	0.16 (3)*	
H12C	0.9147	0.5046	0.2592	0.10 (2)*	

### Atomic displacement parameters (Å^2^)

**Table:**

	*U*^11^	*U*^22^	*U*^33^	*U*^12^	*U*^13^	*U*^23^
S1	0.0161 (4)	0.0178 (4)	0.0162 (4)	−0.0001 (3)	−0.0033 (3)	−0.0018 (3)
F1	0.0214 (9)	0.0162 (9)	0.0177 (9)	0.0042 (8)	0.0002 (7)	−0.0025 (7)
F3	0.0324 (11)	0.0220 (10)	0.0136 (9)	0.0064 (9)	−0.0028 (8)	−0.0005 (8)
F2	0.0306 (11)	0.0234 (10)	0.0145 (9)	0.0082 (9)	−0.0053 (8)	−0.0002 (8)
F4	0.0228 (10)	0.0166 (10)	0.0167 (9)	0.0058 (8)	0.0007 (7)	−0.0035 (7)
O1	0.0318 (13)	0.0227 (13)	0.0157 (12)	−0.0023 (10)	0.0014 (9)	−0.0032 (10)
O2	0.0325 (13)	0.0152 (12)	0.0186 (13)	0.0006 (10)	−0.0001 (9)	−0.0045 (9)
O3	0.0252 (12)	0.0132 (12)	0.0174 (11)	0.0024 (10)	−0.0010 (9)	−0.0040 (9)
O4	0.0336 (13)	0.0209 (12)	0.0129 (12)	−0.0040 (10)	−0.0002 (9)	−0.0019 (9)
O5	0.0320 (13)	0.0196 (13)	0.0181 (13)	0.0015 (11)	0.0003 (9)	−0.0066 (10)
O6	0.0239 (12)	0.0158 (12)	0.0169 (12)	0.0022 (10)	−0.0012 (9)	−0.0040 (9)
O7	0.0265 (12)	0.0270 (12)	0.0151 (11)	−0.0078 (10)	−0.0081 (9)	0.0021 (9)
O8	0.0146 (11)	0.0257 (12)	0.0206 (11)	0.0063 (9)	−0.0047 (8)	−0.0070 (9)
O9	0.0319 (13)	0.0156 (11)	0.0413 (14)	−0.0019 (10)	−0.0131 (11)	0.0043 (10)
O10	0.0191 (11)	0.0268 (12)	0.0179 (11)	0.0033 (10)	−0.0006 (8)	−0.0026 (9)
O11	0.0332 (13)	0.0231 (13)	0.0193 (12)	0.0034 (11)	−0.0085 (10)	−0.0015 (10)
O12	0.0265 (13)	0.0342 (15)	0.0410 (15)	−0.0086 (12)	−0.0158 (11)	0.0045 (11)
O13	0.0365 (14)	0.0257 (13)	0.0207 (12)	0.0012 (11)	0.0017 (10)	−0.0029 (11)
O14	0.0387 (14)	0.0251 (13)	0.0214 (12)	−0.0017 (12)	0.0055 (10)	−0.0055 (10)
O15	0.0355 (14)	0.0229 (12)	0.0177 (12)	0.0002 (11)	−0.0076 (10)	−0.0003 (10)
N1	0.0165 (14)	0.0176 (14)	0.0146 (13)	0.0007 (12)	−0.0010 (11)	−0.0035 (11)
N2	0.0274 (15)	0.0154 (14)	0.0116 (14)	−0.0017 (12)	0.0027 (11)	−0.0027 (11)
N3	0.0270 (16)	0.0161 (15)	0.0130 (14)	0.0065 (12)	−0.0018 (11)	−0.0004 (12)
N4	0.0147 (13)	0.0133 (14)	0.0130 (13)	0.0022 (11)	−0.0018 (10)	−0.0013 (11)
N5	0.0212 (14)	0.0149 (14)	0.0137 (13)	−0.0016 (11)	0.0004 (11)	−0.0014 (11)
N6	0.0257 (15)	0.0142 (13)	0.0108 (13)	−0.0010 (12)	0.0012 (11)	−0.0028 (11)
N7	0.0245 (15)	0.0159 (15)	0.0146 (14)	0.0022 (12)	−0.0012 (11)	−0.0007 (12)
N8	0.0161 (14)	0.0147 (14)	0.0127 (13)	0.0007 (11)	−0.0019 (10)	−0.0025 (11)
C1	0.0164 (16)	0.0156 (17)	0.0201 (18)	−0.0038 (14)	−0.0003 (13)	−0.0047 (14)
C2	0.0128 (15)	0.0193 (17)	0.0129 (15)	−0.0003 (13)	0.0002 (12)	−0.0026 (13)
C3	0.0151 (15)	0.0187 (17)	0.0137 (16)	−0.0033 (14)	−0.0005 (12)	−0.0008 (13)
C4	0.0111 (15)	0.0161 (17)	0.0123 (16)	−0.0014 (13)	−0.0004 (12)	−0.0023 (13)
C5	0.0128 (15)	0.0086 (16)	0.0175 (16)	0.0034 (13)	−0.0030 (12)	0.0010 (12)
C6	0.0150 (16)	0.0142 (16)	0.0153 (16)	−0.0020 (13)	0.0005 (12)	−0.0035 (13)
C7	0.0174 (17)	0.0212 (18)	0.0117 (16)	0.0025 (14)	−0.0044 (12)	0.0017 (13)
C8	0.0114 (15)	0.0161 (17)	0.0178 (17)	−0.0012 (13)	−0.0011 (12)	−0.0024 (13)
C9	0.0111 (15)	0.0142 (16)	0.0149 (16)	−0.0021 (13)	−0.0006 (12)	−0.0002 (12)
C10	0.0141 (15)	0.0113 (16)	0.0164 (16)	−0.0039 (13)	−0.0013 (12)	−0.0025 (12)
C11	0.0183 (16)	0.0120 (16)	0.0152 (15)	0.0035 (14)	−0.0030 (12)	−0.0010 (13)
C12	0.0278 (18)	0.0157 (17)	0.0184 (17)	0.0019 (15)	−0.0005 (13)	0.0005 (13)
C13	0.0199 (16)	0.0160 (16)	0.0178 (16)	0.0006 (13)	0.0013 (12)	−0.0009 (13)
C14	0.0211 (17)	0.0163 (16)	0.0153 (16)	−0.0041 (14)	−0.0031 (13)	−0.0023 (13)
C15	0.0182 (16)	0.0201 (17)	0.0119 (15)	0.0015 (14)	−0.0027 (12)	−0.0016 (13)
C16	0.0180 (17)	0.0194 (17)	0.0158 (16)	−0.0018 (14)	−0.0025 (13)	−0.0027 (13)
C17	0.0239 (17)	0.0163 (17)	0.0114 (15)	0.0006 (14)	−0.0003 (12)	−0.0014 (12)
C18	0.0202 (17)	0.0179 (17)	0.0193 (17)	−0.0006 (14)	0.0024 (13)	−0.0014 (13)
C19	0.0227 (17)	0.0165 (17)	0.0204 (16)	0.0038 (14)	−0.0028 (13)	−0.0001 (13)
C20	0.0182 (16)	0.0172 (18)	0.0163 (17)	−0.0052 (14)	0.0037 (13)	−0.0037 (13)
C21	0.0149 (16)	0.0132 (16)	0.0164 (16)	−0.0044 (13)	−0.0017 (12)	−0.0028 (13)
C22	0.0152 (15)	0.0201 (17)	0.0110 (15)	−0.0028 (14)	−0.0007 (12)	−0.0036 (13)
C23	0.0119 (15)	0.0146 (17)	0.0145 (16)	−0.0010 (13)	−0.0052 (12)	0.0000 (13)
C24	0.0134 (15)	0.0101 (16)	0.0194 (17)	0.0028 (13)	−0.0016 (12)	−0.0024 (13)
C25	0.0118 (15)	0.0190 (17)	0.0145 (16)	0.0000 (13)	0.0015 (12)	−0.0041 (13)
C26	0.0163 (16)	0.0186 (17)	0.0085 (15)	−0.0026 (14)	−0.0034 (12)	0.0025 (13)
C27	0.0115 (15)	0.0117 (16)	0.0194 (17)	0.0003 (13)	−0.0029 (12)	0.0003 (13)
C28	0.0113 (15)	0.0129 (16)	0.0161 (16)	−0.0026 (13)	−0.0024 (12)	−0.0011 (12)
C29	0.0138 (16)	0.0128 (16)	0.0174 (16)	−0.0030 (13)	−0.0012 (12)	−0.0045 (13)
C30	0.0169 (16)	0.0115 (16)	0.0171 (16)	0.0041 (13)	−0.0020 (12)	−0.0002 (12)
C31	0.0223 (17)	0.0154 (16)	0.0179 (16)	0.0034 (14)	−0.0003 (13)	0.0006 (13)
C32	0.0195 (17)	0.0119 (16)	0.0218 (17)	0.0023 (14)	−0.0043 (13)	−0.0032 (13)
C33	0.0234 (17)	0.0155 (17)	0.0142 (15)	−0.0035 (14)	0.0016 (13)	−0.0017 (13)
C34	0.0221 (16)	0.0171 (17)	0.0111 (15)	−0.0017 (14)	−0.0028 (12)	−0.0010 (12)
C35	0.0189 (17)	0.0148 (16)	0.0157 (16)	−0.0027 (13)	−0.0020 (12)	−0.0026 (12)
C36	0.0194 (17)	0.0166 (17)	0.0170 (16)	−0.0010 (14)	0.0000 (13)	−0.0007 (13)
C37	0.0264 (18)	0.0221 (18)	0.0140 (16)	−0.0017 (15)	0.0031 (13)	−0.0029 (13)
C38	0.0314 (19)	0.0203 (18)	0.0204 (17)	−0.0008 (15)	0.0021 (14)	−0.0023 (14)

### Geometric parameters (Å, °)

**Table:**

S1—O9	1.460 (2)	C8—C9	1.431 (4)
S1—O10	1.472 (2)	C9—C10	1.447 (4)
S1—O8	1.487 (2)	C11—C13	1.501 (4)
S1—O7	1.4904 (19)	C11—C12	1.504 (4)
F1—C5	1.368 (3)	C11—H11	1.0000
F3—C26	1.373 (3)	C12—C13	1.503 (4)
F2—C7	1.368 (3)	C12—H12A	0.9900
F4—C24	1.370 (3)	C12—H12B	0.9900
O1—C1	1.223 (3)	C13—H13A	0.9900
O2—C1	1.321 (4)	C13—H13B	0.9900
O2—H6	0.8777	C14—C15	1.520 (4)
O3—C10	1.268 (4)	C14—H14A	0.9900
O4—C20	1.216 (4)	C14—H14B	0.9900
O5—C20	1.325 (4)	C15—C19	1.525 (4)
O5—H9	0.9591	C15—H15	1.0000
O6—C29	1.267 (4)	C16—C18	1.518 (4)
O11—H18	0.8603	C16—C17	1.521 (4)
O11—H14	0.8195	C16—H16	1.0000
O12—H12D	0.9288	C17—H17A	0.9900
O12—H12C	0.9437	C17—H17B	0.9900
O13—H13C	0.8409	C18—H18A	0.9800
O13—H13D	0.8982	C18—H18B	0.9800
O14—H14C	1.0015	C18—H18C	0.9800
O14—H14D	0.8887	C19—H19A	0.9800
O15—H15A	0.9103	C19—H19B	0.9800
O15—H15B	0.8949	C19—H19C	0.9800
N1—C15	1.492 (4)	C20—C21	1.491 (4)
N1—C16	1.501 (4)	C21—C22	1.355 (4)
N1—H5	0.9198	C21—C29	1.445 (4)
N1—H13	0.8432	C22—H22	0.9500
N2—C6	1.370 (4)	C23—C24	1.388 (4)
N2—C14	1.458 (4)	C23—C28	1.423 (4)
N2—C17	1.468 (4)	C24—C25	1.401 (4)
N3—C8	1.356 (4)	C25—C26	1.381 (4)
N3—H2	0.8552	C26—C27	1.376 (4)
N3—H10	0.8946	C27—C28	1.427 (4)
N4—C3	1.339 (4)	C28—C29	1.451 (4)
N4—C4	1.404 (4)	C30—C32	1.489 (4)
N4—C11	1.468 (4)	C30—C31	1.499 (4)
N5—C35	1.493 (4)	C30—H30	1.0000
N5—C34	1.495 (4)	C31—C32	1.508 (4)
N5—H8	0.9005	C31—H31A	0.9900
N5—H12	0.8970	C31—H31B	0.9900
N6—C25	1.393 (4)	C32—H32A	0.9900
N6—C36	1.470 (4)	C32—H32B	0.9900
N6—C33	1.478 (4)	C33—C34	1.504 (4)
N7—C27	1.368 (4)	C33—H33A	0.9900
N7—H1	0.8963	C33—H33B	0.9900
N7—H4	0.9266	C34—C38	1.517 (4)
N8—C22	1.344 (4)	C34—H34	1.0000
N8—C23	1.406 (4)	C35—C37	1.519 (4)
N8—C30	1.466 (4)	C35—C36	1.524 (4)
C1—C2	1.486 (4)	C35—H35	1.0000
C2—C3	1.376 (4)	C36—H36A	0.9900
C2—C10	1.435 (4)	C36—H36B	0.9900
C3—H3	0.9500	C37—H37A	0.9800
C4—C5	1.381 (4)	C37—H37B	0.9800
C4—C9	1.425 (4)	C37—H37C	0.9800
C5—C6	1.413 (4)	C38—H38A	0.9800
C6—C7	1.383 (4)	C38—H38B	0.9800
C7—C8	1.396 (4)	C38—H38C	0.9800
			
O9—S1—O10	112.08 (13)	N1—C16—H16	108.8
O9—S1—O8	109.56 (13)	C18—C16—H16	108.8
O10—S1—O8	108.60 (12)	C17—C16—H16	108.8
O9—S1—O7	109.88 (12)	N2—C17—C16	110.7 (2)
O10—S1—O7	109.60 (12)	N2—C17—H17A	109.5
O8—S1—O7	106.98 (12)	C16—C17—H17A	109.5
C1—O2—H6	106.7	N2—C17—H17B	109.5
C20—O5—H9	106.3	C16—C17—H17B	109.5
H18—O11—H14	99.2	H17A—C17—H17B	108.1
H12D—O12—H12C	90.3	C16—C18—H18A	109.5
H13C—O13—H13D	97.9	C16—C18—H18B	109.5
H14C—O14—H14D	107.3	H18A—C18—H18B	109.5
H15A—O15—H15B	94.0	C16—C18—H18C	109.5
C15—N1—C16	113.8 (2)	H18A—C18—H18C	109.5
C15—N1—H5	107.5	H18B—C18—H18C	109.5
C16—N1—H5	109.8	C15—C19—H19A	109.5
C15—N1—H13	118.6	C15—C19—H19B	109.5
C16—N1—H13	103.6	H19A—C19—H19B	109.5
H5—N1—H13	102.8	C15—C19—H19C	109.5
C6—N2—C14	122.5 (2)	H19A—C19—H19C	109.5
C6—N2—C17	123.6 (3)	H19B—C19—H19C	109.5
C14—N2—C17	111.9 (2)	O4—C20—O5	122.0 (3)
C8—N3—H2	113.4	O4—C20—C21	121.6 (3)
C8—N3—H10	123.2	O5—C20—C21	116.4 (3)
H2—N3—H10	118.9	C22—C21—C29	120.2 (3)
C3—N4—C4	120.4 (3)	C22—C21—C20	118.1 (3)
C3—N4—C11	118.2 (2)	C29—C21—C20	121.6 (3)
C4—N4—C11	120.8 (2)	N8—C22—C21	123.8 (3)
C35—N5—C34	113.0 (2)	N8—C22—H22	118.1
C35—N5—H8	114.5	C21—C22—H22	118.1
C34—N5—H8	104.3	C24—C23—N8	121.7 (3)
C35—N5—H12	113.9	C24—C23—C28	118.8 (3)
C34—N5—H12	118.1	N8—C23—C28	119.4 (3)
H8—N5—H12	90.6	F4—C24—C23	119.1 (3)
C25—N6—C36	120.9 (2)	F4—C24—C25	117.0 (3)
C25—N6—C33	118.8 (2)	C23—C24—C25	123.7 (3)
C36—N6—C33	112.4 (2)	C26—C25—N6	125.5 (3)
C27—N7—H1	117.0	C26—C25—C24	114.8 (3)
C27—N7—H4	122.7	N6—C25—C24	119.6 (3)
H1—N7—H4	112.4	F3—C26—C27	115.2 (3)
C22—N8—C23	119.8 (3)	F3—C26—C25	119.2 (3)
C22—N8—C30	118.9 (2)	C27—C26—C25	125.6 (3)
C23—N8—C30	120.8 (2)	N7—C27—C26	119.4 (3)
O1—C1—O2	121.2 (3)	N7—C27—C28	122.4 (3)
O1—C1—C2	122.7 (3)	C26—C27—C28	118.1 (3)
O2—C1—C2	116.1 (3)	C23—C28—C27	118.4 (3)
C3—C2—C10	119.9 (3)	C23—C28—C29	119.7 (3)
C3—C2—C1	118.1 (3)	C27—C28—C29	121.9 (3)
C10—C2—C1	121.9 (3)	O6—C29—C21	121.1 (3)
N4—C3—C2	123.6 (3)	O6—C29—C28	122.4 (3)
N4—C3—H3	118.2	C21—C29—C28	116.4 (3)
C2—C3—H3	118.2	N8—C30—C32	116.2 (2)
C5—C4—N4	121.8 (3)	N8—C30—C31	118.2 (3)
C5—C4—C9	119.2 (3)	C32—C30—C31	60.6 (2)
N4—C4—C9	119.0 (3)	N8—C30—H30	116.7
F1—C5—C4	119.9 (3)	C32—C30—H30	116.7
F1—C5—C6	116.2 (3)	C31—C30—H30	116.7
C4—C5—C6	123.7 (3)	C30—C31—C32	59.39 (19)
N2—C6—C7	124.4 (3)	C30—C31—H31A	117.8
N2—C6—C5	120.8 (3)	C32—C31—H31A	117.8
C7—C6—C5	114.7 (3)	C30—C31—H31B	117.8
F2—C7—C6	119.6 (3)	C32—C31—H31B	117.8
F2—C7—C8	115.0 (3)	H31A—C31—H31B	115.0
C6—C7—C8	125.4 (3)	C30—C32—C31	59.99 (19)
N3—C8—C7	119.0 (3)	C30—C32—H32A	117.8
N3—C8—C9	123.3 (3)	C31—C32—H32A	117.8
C7—C8—C9	117.6 (3)	C30—C32—H32B	117.8
C4—C9—C8	118.6 (3)	C31—C32—H32B	117.8
C4—C9—C10	120.2 (3)	H32A—C32—H32B	114.9
C8—C9—C10	121.1 (3)	N6—C33—C34	109.9 (2)
O3—C10—C2	120.7 (3)	N6—C33—H33A	109.7
O3—C10—C9	122.6 (3)	C34—C33—H33A	109.7
C2—C10—C9	116.8 (3)	N6—C33—H33B	109.7
N4—C11—C13	116.2 (3)	C34—C33—H33B	109.7
N4—C11—C12	118.0 (3)	H33A—C33—H33B	108.2
C13—C11—C12	60.0 (2)	N5—C34—C33	107.6 (2)
N4—C11—H11	116.8	N5—C34—C38	110.0 (2)
C13—C11—H11	116.8	C33—C34—C38	113.0 (2)
C12—C11—H11	116.8	N5—C34—H34	108.7
C13—C12—C11	59.9 (2)	C33—C34—H34	108.7
C13—C12—H12A	117.8	C38—C34—H34	108.7
C11—C12—H12A	117.8	N5—C35—C37	108.6 (2)
C13—C12—H12B	117.8	N5—C35—C36	109.7 (2)
C11—C12—H12B	117.8	C37—C35—C36	111.1 (3)
H12A—C12—H12B	114.9	N5—C35—H35	109.1
C11—C13—C12	60.1 (2)	C37—C35—H35	109.1
C11—C13—H13A	117.8	C36—C35—H35	109.1
C12—C13—H13A	117.8	N6—C36—C35	110.2 (2)
C11—C13—H13B	117.8	N6—C36—H36A	109.6
C12—C13—H13B	117.8	C35—C36—H36A	109.6
H13A—C13—H13B	114.9	N6—C36—H36B	109.6
N2—C14—C15	109.7 (2)	C35—C36—H36B	109.6
N2—C14—H14A	109.7	H36A—C36—H36B	108.1
C15—C14—H14A	109.7	C35—C37—H37A	109.5
N2—C14—H14B	109.7	C35—C37—H37B	109.5
C15—C14—H14B	109.7	H37A—C37—H37B	109.5
H14A—C14—H14B	108.2	C35—C37—H37C	109.5
N1—C15—C14	107.6 (2)	H37A—C37—H37C	109.5
N1—C15—C19	109.5 (2)	H37B—C37—H37C	109.5
C14—C15—C19	113.3 (2)	C34—C38—H38A	109.5
N1—C15—H15	108.8	C34—C38—H38B	109.5
C14—C15—H15	108.8	H38A—C38—H38B	109.5
C19—C15—H15	108.8	C34—C38—H38C	109.5
N1—C16—C18	108.5 (2)	H38A—C38—H38C	109.5
N1—C16—C17	110.8 (2)	H38B—C38—H38C	109.5
C18—C16—C17	111.0 (2)		

### Hydrogen-bond geometry (Å, °)

**Table:**

*D*—H···*A*	*D*—H	H···*A*	*D*···*A*	*D*—H···*A*
N1—H5···O8^i^	0.92	1.81	2.724 (3)	170.
N1—H5···S1^i^	0.92	2.99	3.860 (3)	158.
N1—H13···O10^ii^	0.84	2.12	2.799 (3)	138.
N1—H13···O12^ii^	0.84	2.60	3.257 (4)	135.
N3—H2···O3	0.86	1.97	2.670 (3)	138.
N3—H10···O11	0.89	2.07	2.965 (3)	174.
N5—H8···O12^iii^	0.90	1.80	2.687 (3)	169.
N5—H12···O8	0.90	1.83	2.722 (3)	174.
N5—H12···S1	0.90	2.80	3.628 (3)	154.
N7—H1···O6	0.90	2.00	2.673 (3)	131.
N7—H4···O15	0.93	2.10	2.997 (3)	162.
O2—H6···O3	0.88	1.70	2.523 (3)	156.
O5—H9···O6	0.96	1.64	2.543 (3)	156.
O11—H18···O13^iv^	0.86	2.06	2.918 (3)	177.
O11—H14···O10^iv^	0.82	2.04	2.846 (3)	169.
O12—H12D···O7	0.93	1.81	2.680 (3)	155.
O12—H12D···S1	0.93	2.68	3.479 (2)	145.
O12—H12C···O13	0.94	1.92	2.748 (3)	146.
O13—H13C···O14	0.84	1.90	2.725 (3)	169.
O13—H13D···O4^v^	0.90	1.95	2.774 (3)	153.
O14—H14C···O1	1.00	1.89	2.834 (3)	157.
O14—H14D···O9^vi^	0.89	1.83	2.715 (3)	170.
O15—H15A···O7^vii^	0.91	1.85	2.748 (3)	168.
O15—H15A···S1^vii^	0.91	2.87	3.680 (2)	150.
O15—H15B···O1^iii^	0.89	2.29	2.979 (3)	134.

Symmetry codes: (i) *x*, *y*+1, *z*+1; (ii) *x*−1, *y*+1, *z*+1; (iii) *x*−1, *y*, *z*; (iv) *x*, *y*, *z*+1; (v) *x*+1, *y*, *z*−1; (vi) *x*, *y*+1, *z*; (vii) *x*−1, *y*+1, *z*.
